# Endometriosis of the skeletal muscular system (ESMS): a systematic review

**DOI:** 10.1186/s12905-023-02184-8

**Published:** 2023-01-26

**Authors:** Hui Ye, Chongyang Shen, Qingli Quan, Mingrong Xi, Lin Li

**Affiliations:** 1 Gynecology Department of West China Second University Hospital, Sichuan University, Chengdu, 610041 China; 2 Key Laboratory of Birth Defects and Related Diseases of Women and Children (Sichuan University), Ministry of Education, Chengdu, 610041 China; 3 Basic Medicine School, Chengdu University of Traditional Chinese Medicine, Chengdu, 611137 China; 4 Guangzhou Women and Children’s Medical Center, Guangzhou Medical University, Guangzhou, 510623 China; 5 Fudan University, Shanghai, 201203 China

**Keywords:** Musculoskeletal, Joint, Endometriosis, Delayed diagnosis, Multidisciplinary, Vascular lymphatic metastasis

## Abstract

**Background:**

Extrapelvic endometriosis occurring at skeletal muscle and joint sites is not rare and is prone to delayed diagnosis and inappropriate treatment. Herein, endometriosis of the skeletal muscular system (ESMS) is systematically reviewed to facilitate early diagnosis and treatment.

**Methods:**

Literature on ESMS published before March 2022 was retrieved from the Ovid Medline and Web of Science databases, and the major clinical data were extracted for descriptive analysis.

**Results:**

A total of 62 studies (78 ESMS cases) met these requirements. The ESMS included the abdominal muscles (50.7%), pelvic floor muscles (11.6%), lower limb muscles (11.6%), hip muscles (8.7%), lumbar muscles (7.2%), joints (5.8%), upper limb muscles (2.9%), and shoulder–neck muscles (1.4%). The age was 34.0 ± 7.2 years (range 17–49 years). Approximately 63.8% of patients had at least one previous pelvic surgery, and 76.8% of local symptoms were related to the menstrual cycle. The course of disease was 29.6 ± 25.4 months (range 0.5–96 months). Only 30.3% of the patients sought initial medical advice from gynecologists, while 69.7% sought initial medical advice from a nongynecological physician. Twenty-seven patients underwent fine-needle aspiration (FNA) under ultrasound or CT monitoring, and only 44.4% (12/27) were confirmed to have endometriosis by FNA tissue pathology. Approximately 47.4% (37/78) of the patients had a normal pelvic cavity appearance. Surgical resection was performed in 92.3% (72/78) of the patients, of whom 88.9% (64/72) underwent complete resection of the lesion (negative surgical margin) and 20.8% (15/72) received postoperative hormone therapy. At 16.7 months of follow-up, 83.3%, 13.8%, 2.9%, and four patients had complete response, partial response, recurrence, and permanent function impairment, respectively.

**Conclusion:**

Endometriosis can occur at almost any site in the musculoskeletal system. For women of reproductive age with catamenial pain or a mass in the musculoskeletal system, endometriosis should be suspected. Fine-needle aspiration can easily lead to missed diagnoses. Surgical resection for negative margins is the main treatment, and permanent impairment of function may occur in a few patients due to delayed diagnosis. Vascular lymphatic metastasis is the most likely mechanism of pathogenesis.

## Background

Endometriosis is considered a systemic disease rather than a disease predominantly affecting the pelvis and is defined as the appearance, growth, and infiltration of endometrial tissue (glands and stroma) outside the uterus, causing repeated bleeding, pain, infertility, and nodules or masses. The incidence of endometriosis is increasing yearly, and its prevalence has been estimated at 190 million women worldwide, given the World Bank’s population estimates for 2017 [1, 2], occurring in approximately 10–15% of women of reproductive age, 50% of women suffering from infertility, and 50–80% of women with pelvic pain [1, 3, 4], costing 70 billion dollars annually in the United States alone [3, 5]. Most ectopic endometrium is confined to the pelvic cavity, including the ovary, pelvic peritoneum, vagino-rectum diaphragm, and uterosacral ligament. Endometriosis can also occur outside the pelvic cavity, with a low incidence, accounting for approximately 12% of endometriosis cases. In theory, endometriosis can occur in all organs of the body, including the gastrointestinal tract, urinary tract, upper and lower respiratory systems, diaphragm, chest, pericardium, umbilical cord, abdominal wall, vulva, brain, and musculoskeletal system [6–8].

Endometriosis of the skeletal muscular system (ESMS) is defined as the presence of endometrial glands or stromal cells in skeletal muscles, bones, or joints. To date, beyond the head muscles, cases with ESMS have been reported in the trunk muscles, extremities muscles, pelvis muscles, and limb joints, including the trapezius muscle [9], deltoid muscle [10, 11], rectus abdominis [12–39], obliquus externus abdominis [40, 41], pyramidalis [42], psoas major muscle and iliopsoas muscle [43–47], piriformis muscle [48–51], internal obturator muscle [52, 53], gluteus muscle [54–59], Levator ani and coccygeus [60, 61], vastus lateralis muscle [62–65], thigh adductor muscle and gracilis [66], biceps femoris muscle [67, 68], soleus and gastrocnemius [69], shoulder joint [70], wrist joint [71], and knee joint [72, 73]. ESMS has highly variable manifestations due to the heterogeneity of lesion location; the symptoms are usually atypical, the pain is often not proportional to the size of the lesion, and sometimes ESMS does not coexist with pelvic endometriosis, which may lead to misdiagnosis or delayed diagnosis, prolonged therapy, or impaired function of the patients [4]. The delayed diagnosis time of endometriosis ranges from 4 to 11 years, with 65% of women initially misdiagnosed [74, 75]. For extrapelvic endometriosis, the misdiagnosis rate can be higher due to its heterogeneity.

**Fig. 1:**
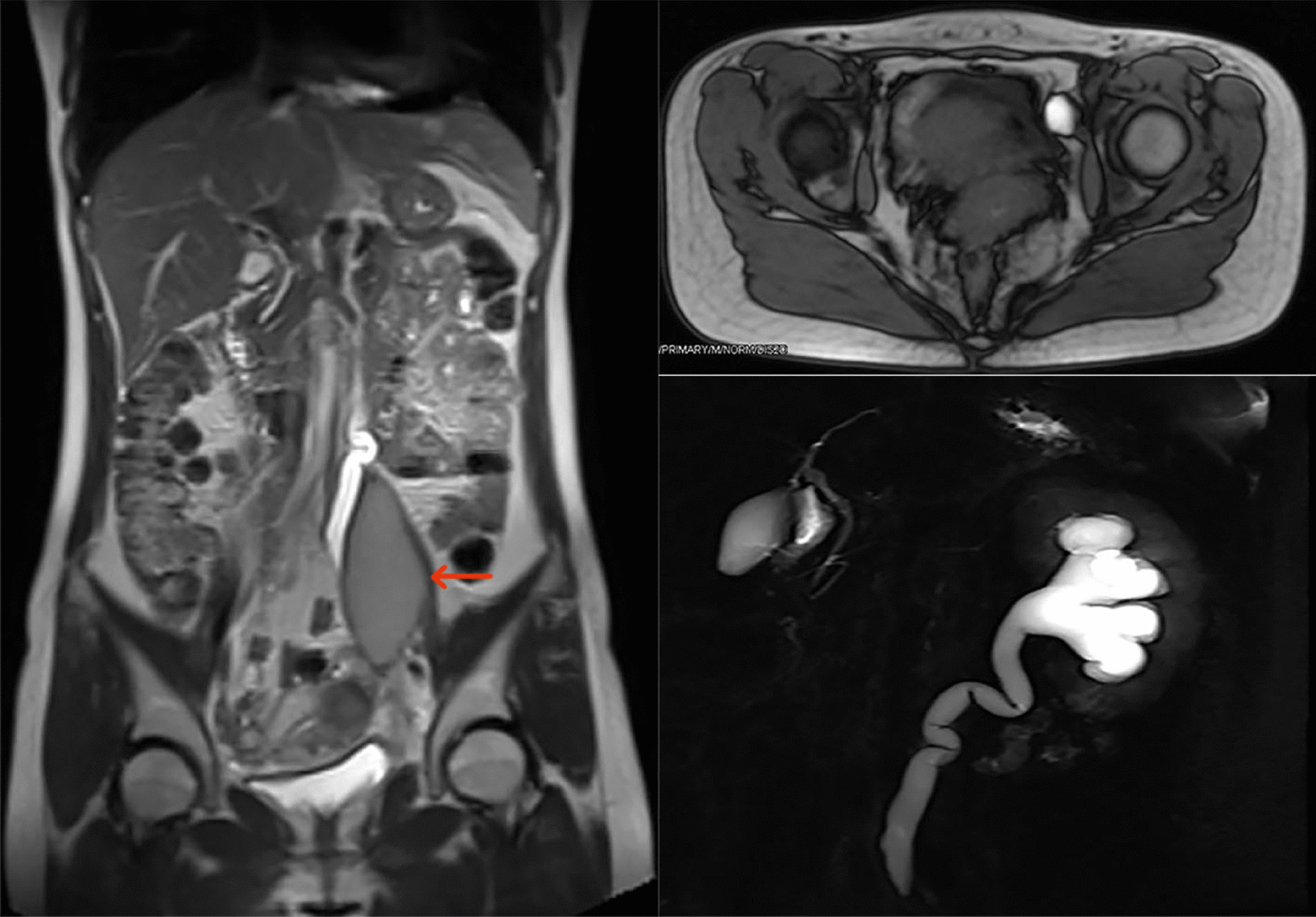
Endometriosis of the left psoas major muscle resulting in left ureter dilation and hydronephrosis

A patient with psoas major endometriosis accompanied by obstructive nephropathy (Fig. 1) was admitted to our department, had previously seen a doctor in three hospitals and four departments and was diagnosed with delayed diagnosis for several years. Although the treatment outcome was satisfactory, the case prompted us to explore how many ESMS cases occurred and how to avoid delayed diagnosis and provide proper treatment. However, the published literature revealed that all ESMS studies to date were case reports or series of reports. To our knowledge, this is the first time that ESMS has been proposed and systematically reviewed. The aim of this systematic review is to recognize the full scope of this disease, facilitate multidisciplinary diagnosis by gynecologists, orthopedists, general surgeons, neurologists, family physicians, orthopedic surgeons, urologists, and even radiologists, allow timely comprehensive treatment, avoid delayed diagnosis and damage to patients, and provide insight into the extrapelvic endometriosis pathogenesis mechanism.

## Methods

All methods were performed in accordance with relevant guidelines. This systematic review was conducted in accordance with the Preferred Reporting Items for Systematic Reviews and Meta-Analyses (PRISMA) guidelines. Data were extracted and analyzed from previously published articles; therefore, ethical approval was not needed.

### Eligibility criteria

The inclusion criteria were as follows: endometriosis lesions that were observed by imaging and surgery, penetration of the muscle tendon sheath, reaching the muscle surface or deep layer or synovium of the joint, and pathological examination revealing endometrial glands and stroma in the skeletal muscle fibers or synovium. Exclusion criteria included subcutaneous tissue endometriosis, skin endometriosis, sciatic nerve endometriosis, or scar endometriosis without muscle invasion. Scar endometriosis after cesarean section with muscle invasion, including rectus abdominis, pyramidalis, obliquus externus abdominis, obliquus internus abdominis, and transversus abdominis, was considered abdominal muscle endometriosis and included. Scar endometriosis after cesarean section without description of muscle invasion in intraoperative observations, imaging examinations or histopathology was considered simple scar endometriosis and excluded. Conference abstracts, reviews, repeated publications, and languages other than English were excluded.

### Information sources

**Fig. 2:**
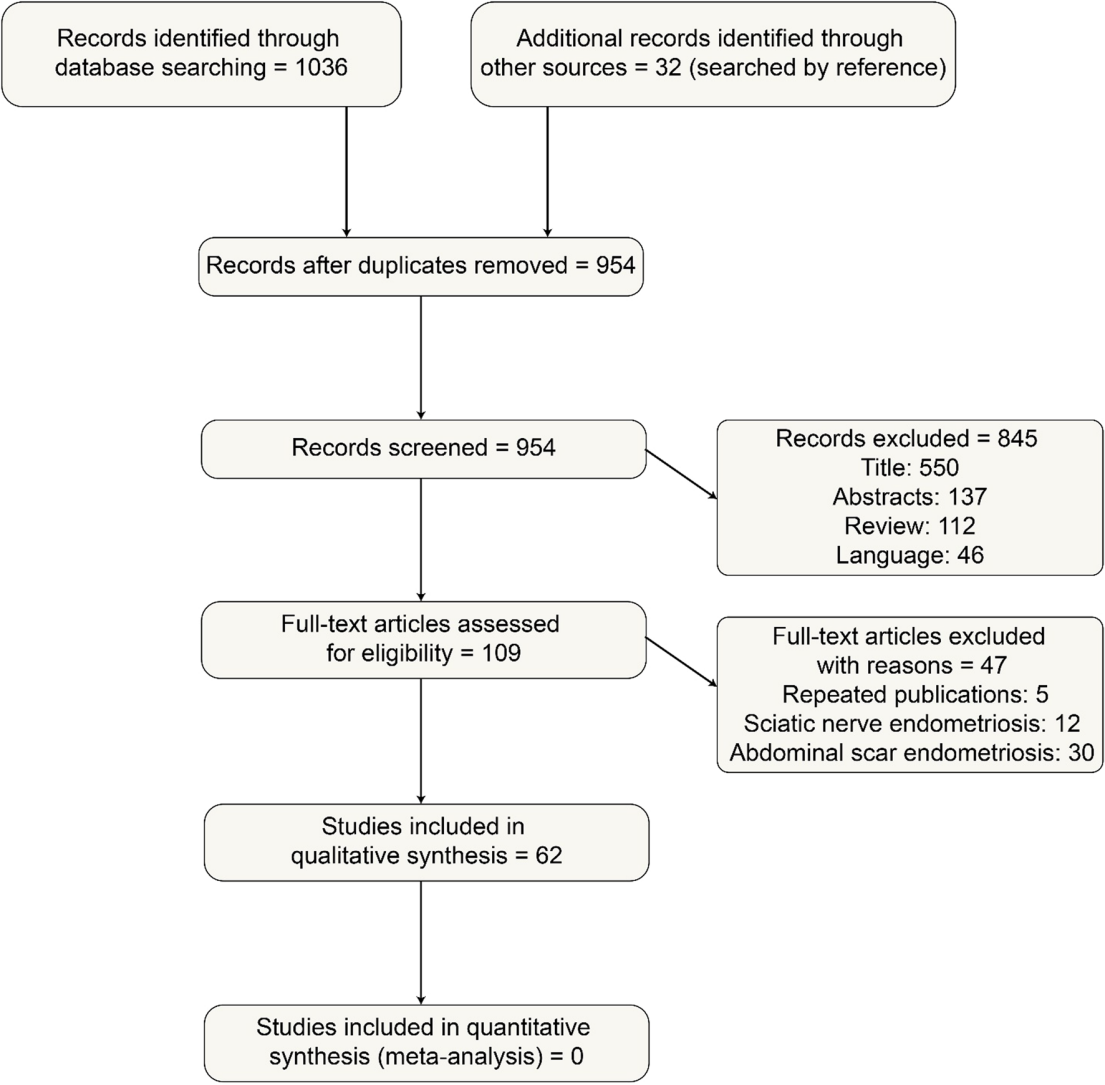
Flowchart of the literature screening process

The literature on ESMS published between 1946 and March 2022 was retrieved from the Ovid Medline and Web of Science databases. Keywords were (“skeletal” or “muscle” or “joint” or “rectus abdominis” or “pyramidal” or “pyramidalis” or “obliquus externus abdominis” or “external oblique muscle” or “obturatus internus” or “obturator Internus” or “gluteus” or “gluteal” or “piriformis” or “piriform” or “levator ani” or “pelvic floor muscle” or “trapezius” or “deltoid” or “psoas” or “iliopsoas” or “multifidus” or “erector spinae” or “paralumbar” or “biceps femoris” or “adductor tight compartment” or “vastus lateralis” or “soleus” or “gastrocnemius” or “limb” or “thigh” or “arthrosis” or “shoulder” or “wrist” or “knee”) and (“endometriosis” or “endometrioma” or “adenomyosis” or “catamenial” or “cyclic” or “periodic”). Some of the original full text was traced through references. A flowchart of the literature screening process is shown in Fig. 2.

### Quality assessment and selection process

The study quality assessment tool (Table 1) was used according to the study design to assess the quality of observational studies (https://www.nhlbi.nih.gov/health-topics/study-quality-assessment-tools). Independent literature screening was performed by two personnel (Chongyang Shen and Qingli Quan); if no agreement was reached by the two reviewers, then it was reevaluated by the third reviewer (Hui Ye). First, the titles and abstracts were screened, and those with uncertainty were checked by reading the full text. Second, the full texts of selected titles and abstracts were carefully read, and supplementary full text retrieval and evaluation were performed based on the bibliography of the second step.

**Table 1:** Quality assessment of case reports or case series included in the systematic review

Author, References	1	2	3	4	5	6	7	8	9	Quality
Leandro [9]	Y	Y	N	NA	Y	Y	Y	NA	Y	Good
Yukitaka [10]	Y	Y	N	NA	Y	Y	Y	NA	Y	Good
Kaur [11]	Y	Y	N	NA	Y	Y	Y	NA	Y	Good
Tanaka [70]	Y	Y	N	NA	Y	Y	Y	NA	Y	Good
Ding [71]	Y	Y	N	NA	Y	Y	NR	NA	Y	Fair
Gabriel [15]	Y	Y	N	NA	Y	Y	Y	NA	Y	Good
Granese [12]	Y	Y	Y	NA	Y	Y	Y	NA	Y	Good
Luca [13]	Y	Y	N	NA	Y	Y	Y	NA	Y	Good
Roberto [14]	Y	Y	N	NA	Y	Y	Y	NA	NR	Fair
Mishin [15]	Y	Y	N	NA	Y	Y	Y	NA	Y	Good
Brian [30]	Y	Y	N	NA	Y	Y	NR	NA	Y	Fair
Karaman [26]	Y	Y	N	NA	Y	Y	NR	NA	Y	Fair
Slaiki [20]	Y	Y	N	NA	Y	Y	Y	NA	Y	Good
Roberge [21]	Y	Y	N	NA	Y	Y	NR	NA	Y	Fair
Ozkan [23]	Y	Y	N	NA	Y	Y	Y	NA	Y	Good
Van Camp [17]	Y	Y	N	NA	Y	Y	NR	NA	NR	Poor
Wasserman [16]	Y	Y	N	NA	Y	Y	NR	NA	NR	Poor
Akhtar [34]	Y	Y	N	NA	Y	Y	NR	NA	Y	Fair
Rani [22]	Y	Y	N	NA	Y	Y	Y	NA	NR	Fair
Coccia [32]	Y	Y	N	NA	Y	Y	Y	NA	Y	Good
Tamiolakis [36]	Y	Y	N	NA	Y	Y	NR	NA	NR	Poor
Sofoudis [19]	Y	Y	N	NA	Y	Y	NR	NA	NR	Poor
Mostafa [24]	Y	Y	Y	Y	Y	Y	Y	NA	Y	Good
Kandil [27]	Y	Y	N	NA	Y	Y	Y	NA	Y	Good
Goker [28]	Y	Y	Y	NA	Y	Y	NR	NA	Y	Fair
Coeman [31]	Y	Y	Y	NA	Y	Y	NR	NA	Y	Fair
Calo [38]	Y	Y	Y	Y	Y	Y	NR	NA	NR	Poor
Barlas [33]	Y	Y	Y	Y	Y	Y	NR	NA	NR	Poor
Toullalan [18]	Y	Y	Y	NA	Y	Y	Y	NA	Y	Good
Ibrahim [39]	Y	Y	N	NA	Y	Y	NR	NA	NR	Poor
Emine [40]	Y	Y	N	NA	Y	Y	NR	NA	NR	Poor
Chiaramonte [41]	Y	Y	N	NA	Y	Y	Y	NA	Y	Good
Crespo [42]	Y	Y	N	NA	Y	Y	NR	NA	NR	Poor
Fangxu [43]	Y	Y	N	NA	Y	Y	CD	NA	Y	Fair
Lingjun [44]	Y	Y	N	NA	Y	Y	Y	NA	Y	Good
Chan [45]	Y	Y	N	NA	Y	Y	Y	NA	Y	Good
Andrade [47]	Y	Y	N	NA	Y	Y	NR	NA	Y	Fair
Bhat [46]	Y	Y	N	NA	Y	Y	NR	NA	Y	Fair
Hickey [48]	Y	Y	N	NA	Y	Y	Y	NA	Y	Good
Olsen [49]	Y	Y	N	NA	Y	Y	Y	NA	Y	Good
Dominguez [50]	Y	Y	N	NA	Y	Y	NR	NA	Y	Fair
Guida [57]	Y	Y	N	NA	Y	Y	Y	NA	Y	Good
Filipa [52]	Y	Y	N	NA	Y	Y	Y	NA	Y	Good
Abraham [54]	Y	Y	N	NA	Y	Y	Y	NA	Y	Good
Volpi [53]	Y	Y	N	NA	Y	Y	Y	NA	Y	Good
Laura [55]	Y	Y	N	NA	Y	Y	NR	NA	NR	Poor
Yekeler [51]	Y	Y	N	NA	Y	Y	Y	NA	Y	Good
Liang [56]	Y	Y	N	NA	Y	Y	Y	NA	Y	Good
Pham [59]	Y	Y	N	NA	Y	Y	Y	NA	Y	Good
Carrasco [61]	Y	Y	N	NA	Y	Y	Y	NA	Y	Good
Claudio [60]	Y	Y	N	NA	Y	Y	CD	NA	Y	Fair
Reddy [58]	Y	Y	N	NA	Y	Y	NR	NA	Y	Fair
Fambrini [66]	Y	Y	N	NA	Y	Y	Y	NA	Y	Good
Pat [63]	Y	Y	N	NA	Y	Y	NR	NA	Y	Fair
Leslie [62]	Y	Y	N	NA	Y	Y	NR	NA	NR	Poor
Pareja [64]	Y	Y	N	NA	Y	Y	NR	NA	NR	Poor
Gitelis [65]	Y	Y	N	NA	Y	Y	CD	NA	NR	Poor
Schlicke [67]	Y	Y	N	NA	Y	Y	Y	NA	NR	Fair
Giangarra [68]	Y	Y	N	NA	Y	Y	NR	NA	Y	Fair
Omero [69]	Y	Y	N	NA	Y	Y	Y	NA	Y	Good
Virendra [72]	Y	Y	N	NA	Y	Y	Y	NA	Y	Good
Jetse [73]	Y	Y	N	NA	Y	Y	NR	NA	Y	Fair

1. Was the study question or objective clearly stated? 2. Was the study population clearly and fully described, including a case definition? 3. Were the cases consecutive? 4. Were the subjects comparable? 5. Was the intervention clearly described? 6. Were the outcome measures clearly defined, valid, reliable, and implemented consistently across all study participants? 7. Was the length of follow-up adequate? 8. Were the statistical methods well-described? 9. Were the results well-described?

*Y*, yes; *N*, no; *CD*, cannot determine; *NA*, not applicable; *NR*, not reported

### Data collection process

The main clinical indicators were extracted for analysis, including publication year, first author, sites, number of cases, age, gestation times, left or right side, surgical history, symptoms, relationship with menstruation, course of the disease, time from onset to the last operation, reason for delayed diagnosis, primary outpatient department, initial diagnosis, preoperative imaging, focal size, fine needle aspiration (FNA), pelvic examination, serum CA125 level, preoperative hormone therapy, surgery, incisional margin, postoperative hormone therapy, follow-up, and prognosis. Prognosis included complete response (pain or mass disappeared completely), partial response (pain or mass was partially gone), recurrence (pain or mass reappeared after symptoms disappeared for 6 months), and sequelae (permanent impairment of function). To investigate whether ESMS is related to pelvic endometriosis, we analyzed the symptomatology, physical examination findings, imaging results, and surgical findings in the literature: 1. Patients who underwent pelvic surgery were assessed based on the surgical findings; 2. For those who had no pelvic surgery, the symptomatology (dysmenorrhea, deep dyspareunia, chronic pelvic pain, and infertility), physical examination findings (tender nodules along the uterosacral ligaments or posterior cul-de-sac, pain or induration without nodules in the rectovaginal septum, uterine or adnexal fixation or fullness), and imaging results (ultrasound or pelvic MRI/CT for assessment of endometriomas, fibroids, adenomyosis, or other adnexal masses) were combined to determine whether pelvic endometriosis existed.

### Synthesis and effect measures

According to the location of the lesion, cases of ESMS were classified as abdominal muscle endometriosis (Table 2), other musculoskeletal endometriosis (Table 3), and joint endometriosis (Table 4). SPSS 24.0 Statistical software (Chicago, IL, USA) was used for data analysis. Qualitative data were used for frequency analysis (numbers/percentage), and quantitative data were used as the mean ± standard deviation or median (range).

**Table 2:** Abdominal muscle endometriosis

Location	Age (yr)	Symptoms	Catamenial or not	Imaging	Therapy	Follow-up (m)	Prognosis
Rectus abdominis [12–39]	34.5 (16–48)	Abdominal mass with pain	Y (32/41) N (7/41) NR (2/41)	US (33/41) CT (15/41) MRI (18/41) Barium meal (1/41)	Excision (40/41) GnRH-a (1/41)	18.1 (4–48)	C (39/41) P (1/41) NR (1/41)
Obliquus externus abdominis [40, 41]	31.5 (25–38)	Mass with pain, aggravated during exercise or menstruation	Y (2/2)	US/CT (2/2)	Excision (2/2) Hormone after surgery (1/2)	6	C (2/2)
Pyramidalis [42]	32	Suprapubic mass with swelling and pain, aggravated during menstruation	Y	US/CT	Excision	NR	NR

*Y*, yes; *N*, no; *NR*, not reported; *US*, ultrasound; *CT*, computed tomography; *MRI*, magnetic resonance imaging; *GnRH-a*, gonadotropin-releasing hormone agonist; *C*, complete remission; *P*, partial remission

**Table 3:** Other musculoskeletal endometriosis

Location	Age (yr)	Symptoms	Catamenial or not	Imaging	Therapy	Follow-up (m)	Prognosis
Trapezius muscle [9]	27	Shoulder swelling	N	NR	Excision	2	C
Deltoid muscle [10, 11]	31 (23–39)	Shoulder mass with pain	N (1/2); Y (1/2)	MRI (2/2) PET (1/2)	Excision (1/2) Dienogest + LNG-IUS (1/2)	46 (20–72)	C
Psoas major muscle and iliopsoas muscle [43–47]	37.6 (28–49)	Asymptomatic (1/5); Dysmenorrhea with low back pain radiating to the limb(3/5)	N (1/5) Y (4/5)	US (4/5) CT (3/5) MRI (3/5) CTA (1/5) PET (1/5) Ureteroscopy (1/5)	Biopsy + GnRH-a (2/5) Excision + GnRH-a (3/5)	2–6	C (3/5) P (2/5)
Piriformis muscle [48–51]	37 (29–45)	Pain in buttocks and thighs radiating to the feet	Y (5/5)	MRI (4/4) US (1/4) PET (1/4) Neurophysiologycal study (1/4)	Excision (2/4) Biopsy + hormone (2/4)	8.3 (6–12)	P (2/4) C (2/4) S (2/4)^#^
Internal obturator muscle [52, 53]	35.5 (32–37)	Periodic medial pain in the leg radiating to the knee	Y (2/2)	MRI (2/2) CT (1/2)	Excision (2/2)	15 (6–24)	C (2/2)
Gluteus muscle [54–59]	34.1 (20–47)	Hip/lower back pain and lower limb numbness with limited movement	Y (3/6); N (1/6); NR (2/6)	MRI (5/6) CT (4/6) US (2/6) PET (1/6) Electromyography (1/6)	Excision + GnRH-a (4/6) Excision (1/6) GnRH-a (1/6)	18.3 (3–60)	C (2/6) P (3/6) NR (1/6) S (2/6)^&^
Levator ani and coccygeus [60, 61]	34 (29–39)	Dysmenorrhea, difficulty in defecation and lumbago during menstruation, radiating to the lower limb	Y (2/2)	MRI (2/2) US (1/2) Colonoscopy (1/2) Urodynamic test/cystoscopy (1/2)	Excision (2/2) COC after surgery (1/2)	6	C (1/2) P and S (1/2)^*^
Vastus lateralis muscle [62–65]	32.3 (24–49)	Lateral thigh mass with pain, menstrual aggravation	Y (3/4); NR (1/4)	MRI (2/4) US (1/4) CT (1/4) PET (1/4)	Excision (3/4) Biopsy (1/4)	6	C (3/4) NR (1/4)
Biceps femoris muscle [67, 68]	30 (25–35)	A painful mass in posterior femoral area	Y (2/2)	CT (1/2) NR (1/2)	Excision (2/2)	7	C (2/2)
Thigh adductor muscle and gracilis [66]	45	Chronic pelvic pain, dysmenorrhea, deep pain in the thigh with difficulty in movement	Y	US /MRI/PET	Partial resection + bilateral oophorectomy	6	C
Soleus and gastrocnemius [69]	30	Dysmenorrhea, progressive swelling and pain in the calf	Y	US/MRI	Excision	4	R

*Y*, yes; *N*, no; *NR*, not reported; *US*, ultrasound; *CT*, computed tomography; *MRI*, magnetic resonance imaging; *PET*, positron emission tomography; *CTA*, computed tomography angiography; *GnRH-a*, gonadotropin-releasing hormone agonist; *COC*, combined oral contraceptives; *LNG-IUS*, levonorgestrel releasing intrauterine system; *C*, complete remission; *P*, partial remission; *R*, relapse; *S*, sequela

^#^minor motor deficits persisted [50]; ^&^permanent muscular damage on her left buttock and functional impairment [57], climbing stairs and getting up from squatting were impaired [59]; ^*^ nerve injury [61]

**Table 4:** Joint endometriosis

Location	Age (yr)	Symptoms	Catamenial or not	Imaging	Therapy	Follow-up (m)	Prognosis
Shoulder joint [70]	47	Periodic pain in right shoulder	Y	NR	GnRH-a	35	C
Wrist joint [71]	23	Intermittent swelling and pain in right wrist	Y	US/MRI	Excision	NR	C
Knee joint [72, 73]	24.5 (17–32)	Knee swelling and pain with menstrual aggravation	Y	X-ray/Lower extremity arteriography (1/2) MRI/ arthroscopy (1/2)	Danazol (1/2) Arthroscopic biopsy + COC + LNG-IUS (1/2)	5	C (1/2) R (1/2)

*Y*, yes; *NR*, not reported; *US*, ultrasound; *MRI*, magnetic resonance imaging; *GnRH-a*, gonadotropin releasing hormone agonist; *COC*, combined oral contraceptives; *LNG-IUS*, levonorgestrel-releasing intrauterine system; *C*, complete remission; *R*, relapse

### Reporting bias assessment

Because all published studies on ESMS were case reports or case series reports, an observational study was conducted. There was a publication bias because only English literature was included. There was also a bias of loss to follow-up as follow-up information was not reported in some studies (Tables 2, 3 and 4).

## Results

A total of 62 valid studies, including 78 ESMS cases, were obtained for analysis after screening. Among them, 44 patients had abdominal muscle endometriosis (Table 2), 30 patients had other musculoskeletal endometriosis (Table 3), and 4 patients had joint endometriosis (Table 4).

### Location

**Fig. 3:**
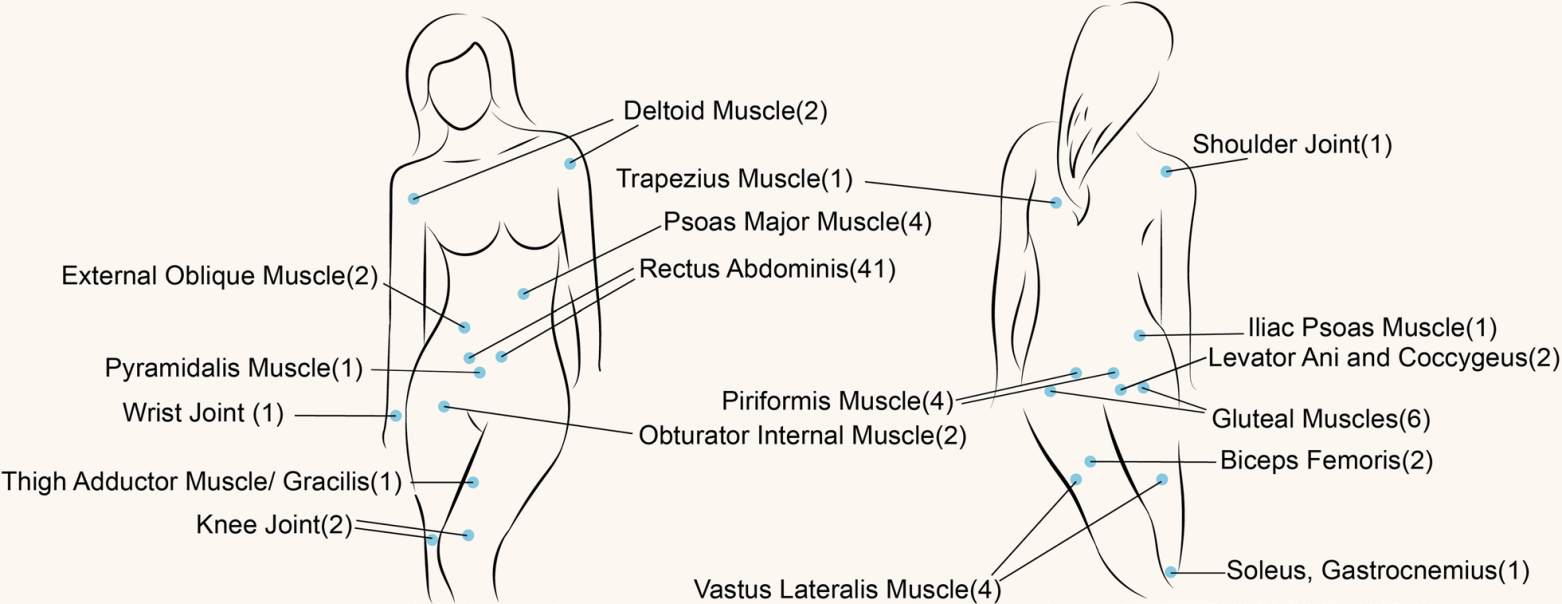
Schematic diagram of the distribution of ESMS: the number in the bracket refers to the number of reported cases

ESMS was reported to occur at the site of almost all skeletal muscles and some joints of the body, as shown in Fig. 3, including the abdominal muscles (50.7%), pelvic floor muscles (11.6%), lower limb muscles (11.6%), hip muscles (8.7%), lumbar muscles (7.2%), joints (5.8%), upper limb muscles (2.9%), and shoulder–neck muscles (1.4%).

### General information

The age of onset ranged from 17 to 49 years, with an average age of 34.0 ± 7.2 years. The incidence rates were 44.9% on the left side, 49.3% on the right side, and 5.8% on both sides. Among the cases, 63.8% (50/78) had at least one previous pelvic surgery, and 36.2% (28/78) had no history of surgery. The course of disease was 29.6 ± 25.4 months (range 0.5–96 months). Only 61.5% (48/78) of the cases reported a history of gravidity, 72.9% (35/48) had a history of parturition, with 71.4% (25/35) through cesarean section and 28.6% (10/35) through natural delivery.

Local symptoms included local pain (53.6%), which mainly manifested as pain at the lesion site (such as joints, limbs, and trunk) during menstruation or several days before and after menstruation, with or without distal limb radiation pain, with or without limited activity, painful lumps (31.9%), painless lumps (7.2%), and local swelling (5.8%). Of the above local symptoms, 76.8% were relevant to the menstrual cycle (symptoms began or worsened during menstruation or several days before and after menstruation), and 23.2% were irrelevant to the menstrual cycle. Systemic symptoms included dysmenorrhea, dyspareunia, chronic pelvic pain, and infertility.

**Fig. 4:**
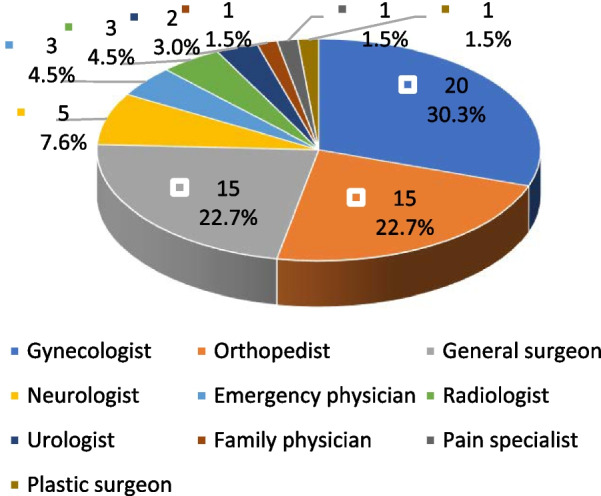
The initial consulted physicians sought by patients with ESMS

The initial consulted physicians were recorded for 66 patients with ESMS. As shown in Fig. 4, only 30.3% (20/66) of patients with ESMS received initial medical advice from a gynecologist, 69.7% (46/66) from nongynecological physicians, including orthopedists (15, 22.7%), general surgeons (15, 22.7%), neurologists (5, 7.6%), emergency physicians (5, 7.6%), radiologists (3, 7.6%), urologists (2, 3.0%), family physicians (1, 1.5%), pain specialists (1, 1.5%), and plastic surgeons (1, 1.5%).

Preoperative imaging examinations included MRI (61.3%), ultrasonography (59.7%), CT (33.9%), and positron emission tomography (PET) (9.7%). Other rare preoperative imaging examinations included arteriography, radiography, barium meals, ureteroscopy, cystoscopy, urodynamics, colonoscopy, arthroscopy, neurophysiology, and electromyography.

The lesion size was 4.1 ± 2.3 cm (range 0.8–12 cm).

Twenty-seven cases received FNA under the guidance of ultrasound or CT, of which only 44.4% (12/27) were confirmed to have endometriosis by FNA tissue pathology.

On physical examination, imaging, and/or surgical exploration, 47.4% (37/78) of patients with ESMS had a normal appearance of the pelvic cavity.

Surgical resection was performed in 92.3% (72/78) of patients, while 7.7% (6/78) received hormone therapy alone. Among patients who underwent surgical treatment, 11.1% (8/72) received hormone therapy before surgery, 88.9% (64/72) underwent complete resection of the lesion (negative surgical margin), 11.1% (8/72) underwent partial resection of the lesion due to difficult resection (positive surgical margin), and 20.8% (15/72) received hormone therapy after surgery. Hormone therapy included gonadotropin-releasing hormone agonist (GnRH-a), combined oral contraceptives (COC), danazol, and progesterone. At 16.7 ± 18.5 months (range 2–72 months) of follow-up, 83.3%, 13.8%, 2.9%, and four patients had complete response, partial response, recurrence, and permanent function impairment, respectively.

## Discussion

The pathogenesis of endometriosis remains controversial, and many theories have been proposed, including menstrual reflux, vascular lymphatic metastasis, iatrogenic implantation, coelomal metaplasia, immune system dysfunction, and stem cells [2–4]. Menstrual reflux usually refluxes to the pelvic cavity; however, the results of this study showed that 47.4% (37/78) of patients with ESMS had a normal pelvic cavity; therefore, menstrual reflux cannot explain the ESMS. Musculoskeletal nerve tissue has a different embryological origin from germ cells and the pelvic peritoneum, so coelomic metaplasia still cannot explain the ESMS. Mignemi et al [76]. believe that distant endometriosis may be explained by lymphatic or hematogenous spreading of endometrial tissue or stem cells, perhaps due to immune dysfunction. The results of this study showed that 63.8% (50/78) of patients with ESMS had at least one previous pelvic surgery, indicating that previous pelvic surgery may be a high-risk factor for ESMS. The most likely explanation for the pathogenesis of ESMS may be metastasis through the endometrium to the musculoskeletal site via vascular lymphatics. Therefore, to minimize the risk of iatrogenic endometrial implantation, invasive gynecological manipulation should be performed during nonmenstrual periods, especially within the week immediately after menstruation. However, 36.2% of patients with ESMS had no history of surgery, indicating that the pathogenesis of ESMS cannot be completely explained by vascular lymphatic metastasis. Accumulating evidence suggests that immune cells, adhesion molecules, extracellular matrix metalloproteinases and proinflammatory cytokines activate/alter the peritoneal microenvironment, creating conditions for the differentiation, adhesion, proliferation and survival of ectopic endometrial cells [77, 78]. The theory of stem cell origin of endometriosis has gained considerable attention in recent years. The strength of the endometrial stem cell theory is that it not only fits the retrograde menstruation model but also explains the pathogenesis of DIE and endometriosis outside the abdominal cavity because stem cells of endometrial origin may enter the angiolymphatic space passively during menstruation and gain entry into the circulation system to find environmentally friendly “soil” for seeding [3]. Canis et al. [79] performed a systematic review, suggesting that local traumatic events may trigger endometriosis. In this systematic review, the traumatic events involved in ESMS could be divided into two parts: Part one is trauma with scarring, including cesarean section, oophorocystectomy, myomectomy, hysterectomy, tubal ligation, pelvic nodule resection, diagnostic laparoscopy, salpingolysis, salpingo-oophorectomy, salpingectomy, inguinal herniorrhaphy, cavernous hemangioma excision, inguinal nodule excision, and appendectomy; Part two is trauma without scarring, including vaginal delivery, dilation and curettage, hysterosalpingography, liposculpture, colonoscopy, appendicitis, and trauma. Local traumatic events may trigger ESMS.

ESMS often presents with atypical symptoms and is often misdiagnosed. Calo PG et al. [38] reported that 2 cases of endometriosis involving the rectus abdominis muscle and made a literature review, they suggested that endometriosis must be included in the differential diagnosis of a symptomatic mass in the abdominal wall in women with and without a surgical history. In the current study, 36.2% of patients with ESMS had no history of surgery, which is consistent with the conclusion of the literature. Chronic fatigue and skeletal muscle pain are both more common in women affected by deep infiltrating endometriosis compared with other subtypes, such as ovarian endometriomas and peritoneal superficial endometriosis [80, 81]. ESMS must be differentially diagnosed from hematoma, lymphatic disease, lymphoma, fibroma, lipoma, abscess, soft tissue sarcoma, and malignancy. Diagnosis is mainly based on symptoms, signs, imaging findings, and sometimes fine-needle aspiration (FNA). For cyclic symptoms associated with menstruation in women of reproductive age, endometriosis should be suspected, and multidisciplinary collaboration should be encouraged to reduce misdiagnoses. Transvaginal ultrasound, with improved sensitivity and specificity, has become the most commonly used imaging tool, while urinary ultrasound can be used to evaluate ureteral compression obstruction. MRI, with additional soft-tissue contrast, is useful for assessing whether the surrounding tissues and organs are invaded [59, 82]. FNA, with histological or cytological tissue obtained, is a theoretically accurate invasive diagnostic procedure [36]. However, in this study, 27 patients with ESMS underwent FNA under ultrasound or CT monitoring, and only 44.4% (12/27) were diagnosed with endometriosis. The low diagnosis rate by FNA was likely related to the small amount of tissue sample, and increasing the number of biopsy samples may help improve the success rate of diagnosis.

Delayed diagnosis is a prominent problem in endometriosis, especially in extrapelvic locations [74, 75]. This study showed that the average course of ESMS was 29.6 months, and the longest course was 96 months. Only 30.3% (20/66) of patients with ESMS sought initial medical advice from a gynecologist, and 69.7% (46/66) sought initial medical advice from nongynecological physicians, including orthopedists (15, 22.7%), general surgeons (15, 22.7%), neurologists (5, 7.6%), emergency physicians (5, 7.6%), radiologists (3, 7.6%), urologists (2, 3.0%), family physicians (1, 1.5%), pain specialists (1, 1.5%), and plastic surgeons (1, 1.5%). The reasons for the delayed diagnosis may be as follows: (1) insufficient attention was given to patients in the early stages of dysmenorrhea. Mothers considered menstruation a negative event, and patients considered dysmenorrhea a normal phenomenon or stigma. (2) The site of ESMS is ubiquitous, the symptoms and imaging manifestations are confusing, and various systemic manifestations, such as the gastrointestinal tract, urinary tract, skeletal muscle, and mental state, may confuse the diagnosis. (3) Patients usually undergo several rounds of treatment in multiple departments before seeking proper treatment. The lack of coherence and integration in medical processes is not beneficial for early diagnosis and treatment. (4) The medical profession is becoming increasingly subdivided, and many medical interns and residents usually pay insufficient attention to the clinical practice of specialties they do not choose. Subsequently, the unfamiliarity with endometriosis has become the norm for many nongynecologists. Therefore, at the medical level, endometriosis should be suspected for catamenial symptoms of reproductive age, and multidisciplinary collaboration is suggested for patients for whom routine treatment is ineffective; at the organizational level, efforts should be made to promote the integration of medical resources, especially in the era of big data. Fortunately, in February 2019, the National Health and Development Commission of China announced the establishment of the National Collaborative Network for Diagnosis and Treatment of Rare Diseases, which is an important step toward the precise diagnosis and treatment of rare diseases. We hope that an international collaborative network will be established in the future.

This study showed that 92.3% (72/78) of the patients with ESMS underwent surgical resection, of whom 88.9% (64/72) underwent complete resection (negative surgical margin), 11.1% (8/72) underwent partial resection (positive surgical margin), and 20.8% (15/72) received postoperative hormone therapy. At 16.7 months of follow-up, 83.3%, 13.8%, 2.9%, and 4 patients had complete, partial, recurrent, and permanent functional impairment, respectively. Surgical excision of lesions is the main treatment for ESMS when medical treatment is ineffective or malignancy cannot be excluded. During the operation, attention should be given to protecting important adjacent organs, such as nerves and the ureter. Multidisciplinary surgery is sometimes necessary to achieve the goal of no residue and minimize side injuries. Bilateral oophorectomy may be considered for perimenopausal patients with repeated recurrence or severe organ function impairment. Preoperative and postoperative adjuvant hormone therapy, including gonadotropin-releasing hormone agonist (GnRH-a), combined oral contraceptives (COC), danazol, and progesterone, may benefit surgery and reduce recurrence. Up to one-third of patients do not respond to first-line therapies because of progesterone resistance or intolerable side effects. Recent literature has shown that GnRH antagonists have resulted in oral drugs (Elagolix has been approved by the FDA, Linzagolix and Relugolix are undergoing clinical trials), which have fewer side effects than other therapies and are a welcome addition in the treatment of endometriosis-associated pain [4]. To prevent recurrence, the ESHRE guidelines strongly recommend long-term administration of postoperative hormone treatment (e.g., combined hormonal contraceptives) for ovarian endometrioma in women not immediately seeking conception but weakly recommend for deep endometriosis [83]. The results of this study suggest that the recurrence rate after complete surgical resection is extremely low, which is insufficient to support the administration of postoperative hormone maintenance therapy for patients with negative resection margins.

## Conclusion

The manifestations of ESMS are diverse due to the heterogeneity of the location. If it is not diagnosed in time, organ function damage can occur in a small number of cases. For women of reproductive age, with catamenial pain, or masses at musculoskeletal sites, ESMS should be highly suspected. Fine-needle aspiration can easily miss ESMS because of the lack of tissue samples. Multidisciplinary cooperation is encouraged to reduce misdiagnoses. Surgical resection with a negative margin is the main treatment, and most of the prognoses are good. Vascular lymphatic metastasis may be one of the most important pathogenic mechanisms. The limitation of this study is that all included studies were case reports or case series reports and observational studies rather than quantitative systematic reviews. Only English literature was included, resulting in publication bias. In addition, some data from the literature were missing. Therefore, the results should be considered with a focus on the salt. The rarity of ESMS presents challenges for the identification and treatment of patients. The true number of ESMS cases worldwide remains unknown. High-quality clinical case reports and collation of epidemiological data favor real-world studies.

## Data Availability

The data used to support the findings of this study are available from the corresponding author upon reasonable request.

## Abbreviations

ESMS: Endometriosis of the skeletal muscular system
FNA: Fine needle aspiration
US: Ultrasound
CT: Computed tomography
MRI: Magnetic resonance imaging
PET: Positron emission tomography
CTA: Computed tomography angiography
CA125: Carbohydrate antigen 125
GnRH-a: Gonadotropin releasing hormone agonist
COC: Combined oral contraceptives
LNG-IUS: Levonorgestrel releasing intrauterine system
